# Blood oxygenation level dependent functional magnetic resonance imaging: current and potential uses in obstetrics and gynaecology

**DOI:** 10.1111/j.1471-0528.2008.01993.x

**Published:** 2008-01

**Authors:** K Vincent, J Moore, S Kennedy, I Tracey

**Affiliations:** a Nuffield Department of Obstetrics and Gynaecology, Oxford University, John Radcliffe Hospital, Headley WayOxford, UK; b Functional Magnetic Resonance Imaging of the Brain Centre, Department of Clinical Neurology, Oxford University, John Radcliffe Hospital, Headley WayOxford, UK

**Keywords:** BOLD fMRI, functional imaging

## Abstract

Blood-oxygenation-level-dependent functional magnetic resonance imaging is a noninvasive technique that has become increasingly popular in the neurosciences. It measures the proportion of oxygenated haemoglobin in specific areas of the brain, mirroring blood flow and therefore function. Here we review how the findings from functional studies impact on areas of gynaecological practice as diverse as chronic pain, continence, and premenstrual dysphoric disorder. Finally we review some of the more novel applications of the technique, such as imaging of pelvic floor function and the effects of hypoxia on the fetus.

## Introduction

Blood oxygenation level dependent (BOLD) functional magnetic resonance imaging (fMRI) is a functional imaging technique that is becoming increasingly popular especially in the neuroscience field of research on human brain function. Here we will give an overview of the physics of the technique and then review some of its current and future applications that are either directly or indirectly applicable to obstetrics and gynaecology.

## Blood oxygenation level dependent fMRI

MRI is a technique commonly used in clinical practice to investigate structural abnormalities. It relies on the different magnetic properties of hydrogen ions in different tissues (e.g. water, fat, bone) to form images with contrast between the tissues, without the need for radioactivity. Although an incredibly useful diagnostic tool in a variety of clinical situations, standard MRI scans are unable to tell us anything about *function*. There are many clinical conditions, such as primary dysmenorrhoea, where structurally the organ appears normal yet symptoms are associated with its function. Previously, the best assessment of function was the patients’ subjective report. However, with the observation of the BOLD response in 1990,^1^ a new era of functional imaging has begun.

**Figure 1:**
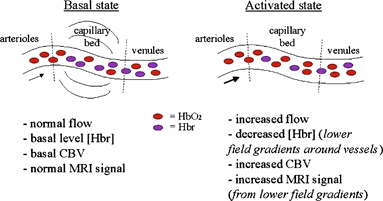
Physiological basis of the BOLD response. HbO2, oxygenated Hb; Hbr, deoxygenated Hb; [Hbr], concentration of Hbr; CBV, cerebral blood volume.

It is well known that metabolic activity uses oxygen; therefore, blood in the vicinity of metabolically active tissues will have a different proportion of oxygenated to deoxygenated haemoglobin (Hb) than that surrounding quiescent tissue. These two forms of Hb have different magnetic properties and this is used as a form of endogenous contrast to allow us to image function rather than structure: BOLD fMRI. For example, activity in a specific brain area is associated with an increase in blood flow to this area, which provides the oxygen and glucose necessary for neuronal activity. In fact, the increase in blood flow exceeds the oxygen requirement and there is therefore an increase in oxygenated Hb relative to deoxygenated Hb in metabolically active areas. This reduction in deoxygenated Hb leads to a reduced magnetic susceptibility and an increased MR signal, which is known as the BOLD response^2^ (Figure 1).

The fact that BOLD fMRI has good spatial (mm) and temporal resolution, is reproducible, and requires no exogenous contrast or radioactivity has made it a popular technique for imaging brain function, both in single-session studies and longitudinal experiments (such as pre- and post-drug treatment). Much work has been performed to investigate the coupling of neuronal activity and local cerebral blood flow, such that we can say with confidence that the BOLD response closely represents neuronal activity.

The development of MR sequences to optimise signal to noise ratio, the increasing availability of high-power magnets (3 or even 7 Tesla), and the introduction of relatively intuitive analysis tools has allowed the technique to be adopted around the world and used in a variety of different fields. Over the last decade, BOLD contrast has also been used to investigate metabolic activity of non-neuronal tissues, some of which will be discussed below.

## Brain imaging studies

### Pain

Pain research is a field that has embraced the use of brain imaging techniques, from early positron emission topography experiments to subtle modern fMRI studies. Both the neural correlates of acute pain^3^ and the aberrant processing that occurs in chronic pain conditions^4^,^5^ have been investigated. These techniques allow either the role of the whole brain, brainstem, or spinal cord to be examined or the contribution of specific brain areas such as the amygdala, insula, or hippocampus to be considered. Furthermore, complex cognitive paradigms can also be included such that the specific effect of modulating, for example, anxiety or attention, can also be seen. When considering clinical pain conditions, experimental stimuli that represent pain in general, for example, thermal stimuli to the inner arm, or a more specifically relevant pain such as balloon distension of the rectum in patients with irritable bowel syndrome (IBS) can be used. Both methods can give interesting and potentially therapeutically useful results. This can be especially useful in conditions where no clear pathological cause exists. Many different pain conditions have been investigated in these studies; however, here we shall consider only two that are gynaecologically relevant.

**Figure 2:**
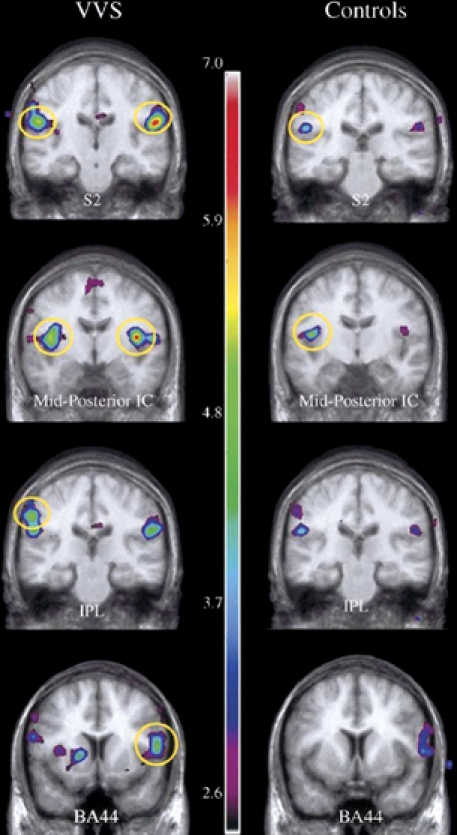
Different brain activation patterns in response to painful pressure in women with VVS and healthy controls. Significant changes are circled in yellow (threshold *t*value = 4.5). Colour bar shows significance. S2, secondary somatosensory cortex; IC, insular cortex; IPL, inferior parietal lobe; BA44, Brodmann’s area 44. Reproduced with permission from Pukall CF, *et al. Pain*2005;115:118–27.^6^

Vulval vestibulitis syndrome (VVS) is a frustrating condition for patients and their doctors, with little to be found on examination or even histology if surgery is undertaken. Yet, an fMRI study has confirmed that the increase in pain and unpleasantness in response to pressure on the vestibule reported by women with VVS (as opposed to pain-free women) is also associated with increased brain activation in response to these stimuli (Figure 2).^6^ This suggests that an augmentation of genital sensory processing exists and points us towards the search for an effective centrally acting drug rather than a local treatment.

IBS is a common cause of pelvic pain, which can show cyclical variations and thus present to the gynaecologist. It has been the subject of a number of fMRI studies (partly because of interest in the concept of the ‘brain–gut axis’ and partly because of the relative ease of accessing the rectum compared with other viscera such as the oesophagus or pancreas) and thus much greater knowledge and understanding exist about this condition than is the case for other visceral pain syndromes. In line with findings from other chronic pain conditions and the concept of visceral hypersensitivity, enhanced brain activation in response to both somatic and visceral^7^,^8^ stimuli has been demonstrated in patients with IBS. Arguably more interesting, however, is the additional finding that subgroups of patients with IBS (diarrhoea or constipation predominant) show differing abnormal endogenous pain inhibitory mechanisms,^9^ suggesting that different treatments may be successful in these groups. Amitriptyline is one of the few proven treatments for IBS, and thus, it is reassuring that patients with IBS treated with this drug, whose symptoms improve, show a normalisation of their brain response to painful rectal stimuli.^10^ This also lends support to the idea that fMRI may be useful as a biomarker in drug development.^11^

Although, to date, no study on dysmenorrhoea has been published, two studies have examined the effects of the menstrual cycle on pain perception.^12^,^13^ Despite the fact that in one of these, there was no significant difference in pain ratings, different patterns of activation in response to pain were seen at different cycle phases in both studies. Therefore, hormonal status may have a role in altering the pain experience, perhaps explaining the frequently observed cyclical variation in symptoms from non-gynaecological organs such as occurs in temporomandibular joint dysfunction^14^ and migraine.^15^

### Continence

The pelvic mechanisms of continence are now relatively well understood, and it is known that in healthy women, this is under higher control. The specific brain areas involved in these mechanisms have now been identified in a series of fMRI studies.

The urge to void has been associated with activity in a network of areas in both the brain and the brainstem and conscious suppression of this urge with strong activity in the superior frontal lobe.^16^ Unsurprisingly, voluntary contraction of pelvic floor muscles in healthy subjects (both men and women) is associated with activation of the supplementary motor area and the primary motor cortex; however, other areas of activation are also seen, presumably reflecting attentive processing and evaluation of visceral sensations. Interestingly, there is no correlation between the bladder volume at the time of pelvic floor contraction and brain activation, and no significant difference in activation patterns between men and women.^17^,^18^

These mechanistic studies in healthy subjects have now been extended into women with stress urinary incontinence to examine the effects of pelvic floor muscle training. After a 12-week programme, more focussed activations were seen in the areas of the primary motor and somatosensory cortices, which represent the lower urogenital tract; these increases also correlated with increased electromyographic activity recorded from the pelvic floor muscles themselves. Furthermore, reduced activity was seen in areas associated with emotional arousal, suggesting that these changes represent more than just pure motor learning.^19^ Women with urge incontinence have also been shown to have abnormal brain responses to bladder filling, with disruption of the connections seen in healthy women and activations thought to represent recruitment of alternative pathways to maintain bladder control.^20^

Studies such as these can therefore inform clinical practice by validating current treatments and by suggesting possible combinations of, for example, physiotherapy, pharmacological, and psychological treatments. A more detailed understanding of the role of the brain, brainstem, and spinal cord in continence mechanisms may also help in the development of novel treatments for urinary incontinence secondary to brain or spinal cord injury.

### Premenstrual dysphoric disorder

Premenstrual dysphoric disorder (PMDD), although a psychiatric diagnosis, frequently presents to the gynaecologist as part of a spectrum of menstrually related disorders or is discussed in the context of hormonal treatment of other symptoms. There is no evidence that abnormal levels of hormones occur in this condition (e.g. unlike in depression associated with hypothyroidism); instead, it appears that some women are more sensitive to the mood-destabilising effects of these hormones.^21^ Comparing women with PMDD to asymptomatic women in both pre- and postmenstrual phases, clear differences can be seen in the fMRI response to emotional words. In the premenstrual phase, women with PMDD showed greater activation in certain areas in response to negative words (particularly the amygdala) and a decreased activation to positive words; furthermore, they failed to activate areas associated with the control of emotional responses.^22^ Other studies have demonstrated an increased excitability of the amygdala in the presence of progesterone in healthy women,^23^ an area known to be involved in anxiety and memory. These results suggest possible mechanisms whereby hormones could influence mood. Of course, hormone-related changes in mood do not just occur in PMDD; postnatal depression and puerperal psychosis are other examples where an understanding of the mechanisms involved would be useful.

Thus, it may be possible to identify those women who are potentially at risk of developing a mood disorder in response to hormonal changes and to establish potential new therapeutic targets. Furthermore, a number of mood-altering/stabilising drugs also affect the hypothalamic–gonadal axis and thus alter hormonal status and reproductive function.^24^ An understanding of the interactions between hormones and mood would inform sensible choice of hormone replacement therapy (HRT) in these patients if it were to be desired.

### Postnatal bonding and parenting behaviour

Bonding between new parents and their baby is something we all know to be important, and in our day-to-day practice, we take great care to identify antenatally those women we fear may have difficulties bonding and try to prevent traumatic intrapartum and postnatal events from interfering with this important process. However, the neural processes involved in parental behaviour and why these may go wrong in unexpected cases are only just beginning to be unravelled. Animal (both rat and non-human primate) studies have confirmed the importance of several hormones (oxytocin, dopamine, prolactin, and estrogen) in the development of this behaviour and suggested candidate brain areas that might be involved. However, it is only recently, with the use of functional imaging, that these networks have been visualised in humans. Perhaps unsurprisingly, it appears that brain circuits involved in parenting behaviour have many similarities with areas regulating other social attachments. Furthermore, these circuits can be activated by both auditory (baby crying) and visual (pictures of babies’ faces) stimuli and, particularly in mothers, to a greater extent when these stimuli are from their own rather than another baby. Components of both empathy and reward circuits also appear to be involved in normal healthy parenting responses in humans.^25^

A number of pathological behaviours such as addiction, anxiety, and mood disorders are associated with abnormal function in the circuits identified in these studies and with disruption of the normal processes of bonding. Thus, it may be that knowledge of conditions or personality traits with similar activation patterns may help us to identify more accurately those at risk of bonding issues and subsequent child neglect. Furthermore, if fMRI studies are able to identify which infant-related stimuli are best able to activate these ‘parenting circuits’, these stimuli could then be used as a component of psychological therapies to enhance emotional bonding between new parent and baby in those who are struggling.

### Effects of hormones on cognitive function

A perceived alteration in cognitive function is a common complaint of perimenopausal and menopausal women and of some women during pregnancy and premenstrually. To what extent could changes in steroid hormone concentrations be responsible for this?

The direct effect of progesterone on amygdala function already discussed may explain the impairment of memory associated with the premenstrual phase and pregnancy,^23^ and cognitive function has certainly been shown to decline around the time of and after the menopause. A number of studies have suggested a neuroprotective effect of estrogen therapy, with a reduced risk of dementia and less cognitive ageing.^26^,^27^ However, other work has suggested that there may be a specific window when these benefits are seen, as in older postmenopausal women such effects are not demonstrated. fMRI allows a more specific investigation of the brain areas influenced by hormonal treatments rather than relying on the interpretation of results of tests of cognitive function. Even in healthy, premenopausal women, the ability to perform language tasks is significantly influenced by hormonal status,^28^,^29^ and prefrontal cognitive tasks, especially verbal recall, are improved by estrogen replacement in postmenopausal women.^30^,^31^ It had been suggested that the beneficial effect seen in perimenopausal women was in fact mediated by the reduction in hot flushes and improved sleep leading to improved functioning. However, fMRI studies have confirmed that this is not the case.^31^ Small studies have also suggested that specific preparations of HRT may have differing effects on cognitive function.^32^ If larger studies confirm this finding, this may be a further factor to take into account when weighing up the risks and benefits of HRT for an individual woman.

## Imaging of non-brain organs

### Pelvic organs

Although originally developed for functional imaging of the brain, in the last decade the technique of BOLD MRI has been used to evaluate perfusion and hypoxia in a number of other organs, including the heart,^33^ kidney,^34^ and muscles.^35^ As these new applications are in their infancy, most studies are evaluating their utility in healthy tissues, and as yet, very few clinical studies have translated these techniques into patients. This is in contrast to brain imaging, where the neural correlates of a huge variety of functions have been elucidated in healthy subjects and then subsequently compared with patients with a variety of relevant pathologies.

In the myometrium, it has been shown that the two layers (outer and junctional zone) function differently, and, as would be expected, their function varies across the phases of the menstrual cycle.^36^ It should therefore be possible to investigate a number of pathological conditions including dysmenorrhoea, adenomyosis, and perhaps even unexplained infertility using this technique. A greater understanding of the functional role of the myometrium in these conditions may suggest novel therapies.

As good results have been obtained imaging skeletal muscle elsewhere in the body, another obvious site to consider would be the pelvic floor, where other functional imaging techniques, such as cine-MRI, have already been used in women with prolapse.^37^ As can be seen from Figure 1, any stimulus that causes oxygen consumption via metabolic activity can be detected by changes in the BOLD signal. BOLD MRI could therefore be useful to identify abnormal contractile areas such as those that occur in pelvic floor tension myalgia, a cause of pelvic pain and dyspareunia.^38^ Other possible applications include investigating areas of relative hypoxia or hyperperfusion within the pelvis as has been hypothesised to occur in primary dysmenorrhoea and in pelvic venous congestion.

### Fetus

MRI is increasingly being used for prenatal diagnosis when the information provided by ultrasound is insufficient;^39^ however, recent work has suggested that BOLD fMRI may also have a role in obstetrics.

**Figure 3:**
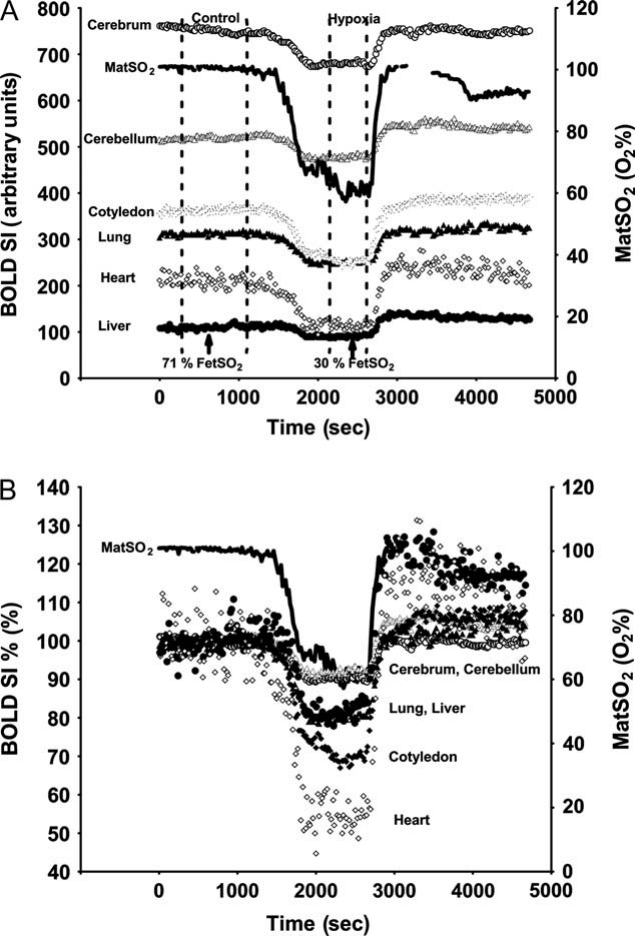
Effect of maternal hypoxia on fetal organ oxygen saturation and BOLD signal. Scatter plots of data from a single animal during an initial control period; hypoxia after reduction of maternal oxyhaemoglobin saturation (MatSO_2_) and recovery. (A) Time course of absolute BOLD signal intensities (BOLD SI); (B) time course of normalised BOLD signal intensities (BOLD SI%). FetSO_2_, fetal oxyhaemoglobin saturation. Reproduced with permission from Wedegartner U, *et al. Radiology*2006;238:872–80.^43^

The delivery of oxygen to tissues is crucial to the developing fetus. Both *in utero*and postnatal cord blood samples have shown growth-restricted fetuses to be hypoxic and acidotic when compared with normally grown fetuses.^40^,^41^ However, a noninvasive test to monitor fetal oxygenation or identify those fetuses at risk of hypoxia and subsequent growth restriction does not exist. It has been shown that maternal oxygen breathing in women in the third trimester significantly increases the MR signal from the fetal liver,^42^ suggesting that BOLD fMRI may be a suitable technique to use. This has been investigated further in animals (although not yet in humans). It can be seen from Figure 3 that in sheep, maternal hypoxia is associated with fetal hypoxia and a differential reduction in BOLD signal from a number of fetal organs.^43^ As would be expected, the reduction in perfusion of brain tissues was less than that seen in the lung, heart, and cotyledon and the fetal oxygen saturation correlated significantly with the BOLD signal. The larger size of the human placenta (as opposed to small sheep cotyledons) should make the assessment of placental perfusion more feasible and may allow the detection of abnormalities earlier than currently available methods. Hence, fetal BOLD MRI may develop into a tool to noninvasively identify and then monitor organ perfusion in the fetus at risk.

**Figure 4:**
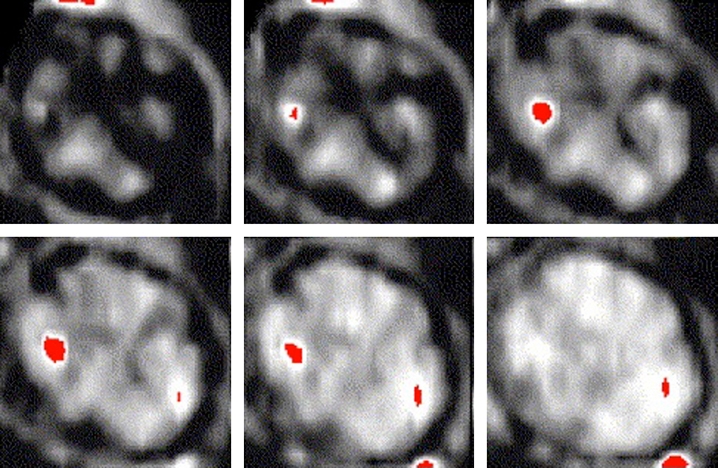
The activation map in response to acoustic stimuli for a single fetus. Reproduced with permission from Gowland P, Fulford J. *Exp Neurol*2004;190:22–7.^44^

Very little is currently known about the development of fetal brain function and a better understanding of this area could inform the management of complicated pregnancies. A number of studies have investigated structural brain development (e.g. cortical folding and myelination) with MRI, but it is only in the last few years that fetal brain function has started to be investigated. Brain activation has now been shown in human fetuses in response to both auditory and visual stimuli (Figure 4) using similar experimental paradigms to those used in adults.^44^ This is a more objective measure than fetal movements or heart rate changes in response to stimuli. By examining women of varying gestation, the age at which these responses develop could be identified. Furthermore, the effect of antenatal hypoxia on the development and maintenance of these pathways could also be assessed. In combination with structural MRI, fetal fMRI may have an important role in neurodevelopmental research.

Studies in rats have been able to demonstrate placental hypoxia with BOLD MRI after giving cloprostenol, a prostaglandin F_2α_ analogue^45^. Thus, this technique could also be useful in the investigation of drug effects on placental perfusion in animals without the need for invasive monitoring. Brain imaging using cognitive paradigms in children and adults who were exposed to certain drugs *in utero*may also help us understand in more detail the effects of these drugs on brain development.^46^

## Conclusions

BOLD fMRI is a technology used widely in many other specialties, both for basic science research and as a clinically applicable tool. Here we have described a number of ways in which it could be applicable to obstetricians and gynaecologists. These range from brain imaging work looking at higher control of functions in healthy women (such as continence) to aberrant processing of information in disease states (such as chronic pain and PMDD), to imaging of other organ systems in both health and disease, in women and their fetuses. Combining information obtained from structural and functional imaging methods is particularly powerful, and by using such complimentary techniques, our knowledge of both physiology and pathophysiology can be greatly enhanced. Novel applications such as fetal monitoring have the potential to make important contributions both to our understanding and our clinical practice.

## Disclosure of interests

No conflict of interests is declared by any author.

## Contribution to authorship

K.V. wrote the manuscript, and J.M., S.K., and I.T. contributed to the final version.

## Funding

K.V. is supported by a research grant from Pfizer.▪
